# Correction: A sensitive OFF–ON–OFF fluorescent probe for the cascade sensing of Al^3+^ and F^−^ ions in aqueous media and living cells

**DOI:** 10.1039/d0ra90067b

**Published:** 2020-06-25

**Authors:** Lingjie Hou, Wenting Liang, Chenhua Deng, Caifeng Zhang, Bo Liu, Shaomin Shuang, Yu Wang

**Affiliations:** Department of Chemistry, Taiyuan Normal University Jinzhong 030619 P. R. China; Department of Chemistry, Institute of Environmental Science, Shanxi University Taiyuan 030006 P. R. China liangwt@sxu.edu.cn wangyu1168@sxu.edu.cn; Humic Acid Engineering and Technology Research Center of Shanxi Province Jinzhong 030619 P. R. China; National Institutes for Food and Drug Control Beijing 100050 P. R. of China

## Abstract

Correction for ‘A sensitive OFF–ON–OFF fluorescent probe for the cascade sensing of Al^3+^ and F^−^ ions in aqueous media and living cells’ by Lingjie Hou *et al.*, *RSC Adv.*, 2020, **10**, 21629–21635, DOI: 10.1039/D0RA02848G.

The authors regret that an incorrect version of Fig. 4 was included in the original article. The correct version of Fig. 4 is presented below.

**Fig. 4:**
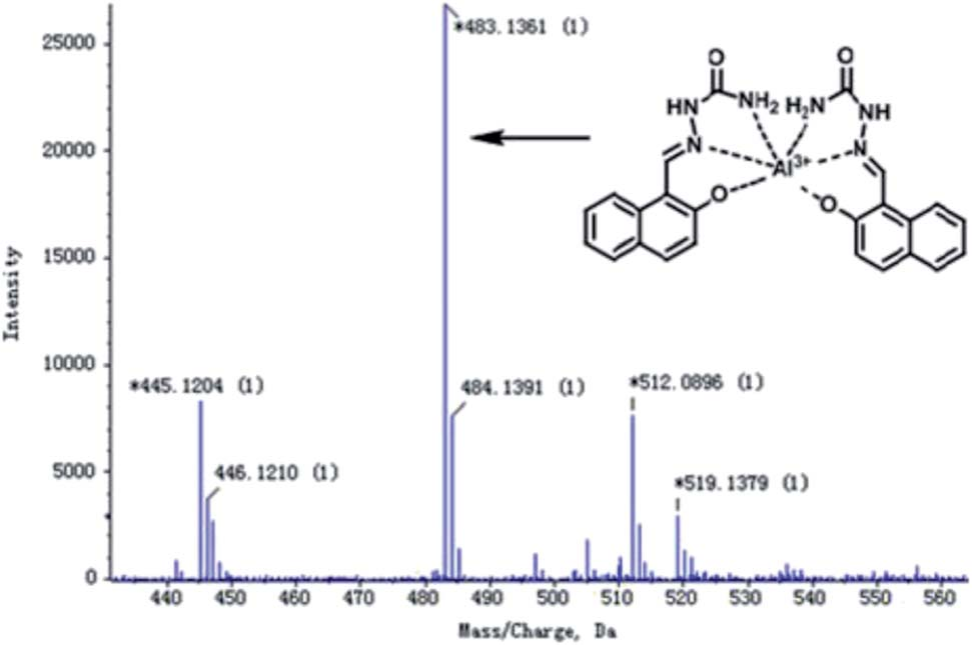
The ESI-MS spectrum of Al^3+^–HNS complex.

The Royal Society of Chemistry apologises for these errors and any consequent inconvenience to authors and readers.

## Supplementary Material

